# β-Catenin Regulates Glycolytic and Mitochondrial Function in T-Cell Acute Lymphoblastic Leukemia

**DOI:** 10.3390/biomedicines13020292

**Published:** 2025-01-24

**Authors:** Ling Zhang, Yu Zhao, Shuoting Wang, Jian Zhang, Xiaohui Li, Shuangyin Wang, Taosheng Huang, Jinxing Wang, Jiajun Liu

**Affiliations:** 1 Department of Hematology, The Third Affiliated Hospital of Sun Yat-sen University, Guangzhou 510630, China; zhangl389@mail.sysu.edu.cn (L.Z.); wangsht39@mail2.sysu.edu.cn (S.W.); lixxiaoh0831@163.com (X.L.); 2 Department of Hematology, The Third Affiliated Hospital of Southern Medical University, Guangzhou 510630, China; zhyhg0203@163.com (Y.Z.); wangshuangyin1998@163.com (S.W.); 3 Guangzhou Institutes of Biomedicine and Health, Chinese Academy of Sciences, Guangzhou 510530, China; shuifen1983@163.com; 4 The Medicine and Biological Engineering Technology Research Center of the Ministry of Health, Guangzhou 510663, China; tsh_huang@sina.cn; 5 Department of Pathology Technique, Guangdong Medical University, Dongguan 523808, China

**Keywords:** β-catenin, T-cell acute lymphoblastic leukemia, glycolysis, mitochondrial function

## Abstract

**Background:** T-cell acute lymphoblastic leukemia (T-ALL) is an aggressive hematological malignancy characterized by a poor prognosis. β-catenin is implicated in the progression of T-ALL, yet the precise mechanisms of β-catenin involvement in the pathogenesis of T-ALL, particularly concerning metabolic processes, remain inadequately elucidated. **Methods:** A β-catenin knockout cell line was generated in the human leukemic cell line Jurkat using the CRISPR-Cas9 technique. Subsequently, assays were performed to evaluate cell proliferation, apoptosis, and metabolic activity. Comparative transcriptomic analysis was conducted between control cells and β-catenin knockout cells. Finally, a mouse xenograft model was employed to assess whether β-catenin knockout attenuates tumor growth and infiltration in vivo. **Results:** The deletion of β-catenin significantly inhibited proliferation and induced apoptosis. Additionally, the silencing of β-catenin led to the inhibition of glycolysis and a reduction in both mitochondrial mass and membrane potential. These results indicate that β-catenin may play a crucial role in regulating cell proliferation and apoptosis through the modulation of glycolytic activity and mitochondrial function in T-ALL. **Conclusions:** In summary, our findings uncover a novel mechanism by which β-catenin influences glycolysis and mitochondrial function in the progression of T-ALL, thereby identifying a potential therapeutic target for patients with relapsed T-ALL.

## 1. Introduction

T-cell acute lymphoblastic leukemia (T-ALL) is an aggressive hematologic malignancy, seen in about 10–15% of children with ALL and 25% of adults with ALL, with a higher incidence in males [1,2]. Despite advancements in treatment modalities, including intensive chemotherapy regimens, stem cell transplantation, and targeted therapeutic agents, the outcomes of relapsed or refractory (R/R) T-ALL remains dismal [3]. Thus, there is an urgent need to explore potential molecular targets implicated in the relapse and progression of T-ALL.

T-ALL occurrence is closely associated with aberrant lymphocyte proliferation, differentiation and apoptosis [4]. The Wnt/β-catenin signaling pathway is crucial in modulating these processes, as well as lymphocyte migration and stemness [5]. The β-catenin protein, a critical component of the Wnt signaling pathway, is implicated in leukemogenesis and progression, the maintenance of the progenitor cell pool in T-ALL [6], and experimental knockout of β-catenin-induced apoptosis in T-ALL cell lines [7]. Furthermore, β-catenin also plays a crucial role in leukemia cell proliferation and survival [8].

Cancer metabolic reprogramming is associated with resistance tumor cells in a harsh environment [9]. Herbst et al. analyzed KEGG pathways in colorectal cancer, revealing that β-catenin regulates insulin signaling and influences the metabolism of the actin cytoskeleton [10]. Furthermore, the knockout of β-catenin in breast cancer cell line (MCF-7) resulted in the downregulation of proteins involved in carbohydrate metabolism and the tricarboxylic acid cycle (TCA), alongside the upregulation of proteins associated with lipid metabolism [11]. These findings underscore the pivotal role of β-catenin in the modulation of metabolic processes and energy homeostasis. However, the involvement and metabolic regulation by β-catenin in T-ALL cells remain poorly understood. This study assessed the impact of β-catenin on glycolysis and mitochondrial function in T-ALL. Our study aimed to elucidate the effect of β-catenin in the progression of T-ALL cells and explore its role in T-ALL metabolism. Specifically, we demonstrate that β-catenin knockout in T-ALL cell line induces metabolic reprogramming, which is characterized by alterations in mitochondrial function and glycolysis.

## 2. Materials and Methods

### 2.1. Cell Culture

Human T-cell lines (Jurkat) were obtained from the American Type Culture Collection (Manassas, VA, USA), and cultured in Roswell Park Memorial Institute (RPMI) 1640 medium (Gibco, GrandIsland, NE, USA) supplemented with 10% fetal bovine serum (FBS, Gibco).

### 2.2. Plasmid Construction and Lentivirus Infection

LentiCRISPR v2-containing sgRNA targeting CTNNB1 (β-catenin) was applied to construct β-catenin knockout cells (Jurkat). The sgRNA sequences were seen as follows: GAAGGTTATGCAAGGTCCCAG. Then, β-catenin knockout cells were screened with puromycin (Thermo Fisher Scientific, Waltham, MA, USA).

### 2.3. Cell Proliferation Assay

A total number of 10,000 Jurkat cells were cultured in 100 μL of medium in 96-well plates for 24, 48, and 72 h. Subsequently, 10 μL Cell Counting Kit-8 (CCK8) reagent (APExBIO, Houston, TX, USA) was added to each well, followed by a 4 h incubation period. Optical density was detected at a wavelength of 450 nm using a spectrophotometric plate reader (BioTek, Burlington, VT, USA). In addition, cell proliferation was also assessed using the carboxyfluorescein succinimidyl ester (CFSE) probe. Specifically, the cells were labeled with CFSE at a final concentration of 5 µM for 30 min at 37 °C in an incubator and then cultured in 6-well plates. After 48 h, the cells were harvested, washed, and subjected to analysis via flow cytometry.

### 2.4. Cell Apoptosis Assay

Cells were harvested, washed twice by phosphate-buffer saline (PBS), and resuspended in a binding buffer. Then, the cells were stained with 5 μL annexin V-FITC and 5 μL propidium iodide (PI) staining solution (Vazyme, Nanjing, China) for 15 min at room temperature in the dark and analyzed using flow cytometry.

### 2.5. Glucose Measurement

The assessment of glucose uptake was conducted utilizing the 2-NBDG probe. Initially, cells were harvested and washed twice by PBS, cultivated in RPMI-1640 medium without glucose in 6-well plates. Then, they were incubated with 2-NBDG in the dark for 1 h at 37 °C and collected, washed, and analyzed via flow cytometry. Glucose consumption was quantified using the Glucose Assay Kit (BC2500, Solarbio, Beijing, China). Briefly, the culture medium was collected, and glucose levels were measured following the manufacturer’s protocol.

### 2.6. Lactate Level Measurement

The lactate concentration secreted by Jurkat cells into the culture medium was quantified utilizing the Lactate Assay Kit (DOJINDO, Kumamoto, Japan). Briefly, a total of 1 × 106 cells were seeded in RPMI-1640 medium devoid of phenol red within a 12-well plate. Following a 4 h incubation period, the cell culture medium was collected. Lactate concentrations in both control and β-catenin knockout cells were determined by measuring the optical density at 490 nm and were calculated based on a standard curve. The relative lactate level was normalized to protein concentration.

### 2.7. Detection of ROS Levels

Reactive oxygen species (ROS) levels were measured utilizing the dihydroethidium (DHE) probe. The cells were harvested and washed by PBS twice. Then, they were incubated with the DHE probe at a final concentration of 10 µM in the dark for 30 min at 37 °C. Following incubation, they were collected, washed, and subjected to flow cytometry analysis. Additionally, cellular imaging was performed using a fluorescent microscope (Olympus, Tokyo, Japan).

### 2.8. Measurement of Mitochondria Membrane Potential

The cells were harvested and washed by PBS twice, and stained at 37 °C with 5 µM JC-1 for 30 min. The mitochondrial membrane potential changes were detected using flow cytometry. Post-acquisition analysis was conducted using FlowJo V10 software. Imaging of the cells was obtained using a fluorescent microscope (Olympus, Tokyo, Japan).

### 2.9. Intracellular Calcium Concentration Assay

The cells were collected and washed twice by D-Hanks buffer (without Ca^2+^ and Mg^2+^), and then stained with 2 μM Fluo-4 AM at room temperature for 30 min. The fluorescence intensity was detected using flow cytometry, and imaging of the cells was obtained utilizing a fluorescent microscope (Olympus, Tokyo, Japan).

### 2.10. Measurement of Mitochondria Content

The cells were collected and washed twice by RPMI 1640, then stained at room temperature with MitoTracker for 30 min. The fluorescence intensity was detected using flow cytometry, and the cells were photographed using a fluorescent microscope (Olympus, Tokyo, Japan).

### 2.11. Measurement of Adenosine Triphosphate (ATP) Levels

We collected and lysed the cells using the reaction buffer. Then, we collected the supernatant to measure the ATP levels on the basis of the protein concentration, following the manufacturer’s instructions.

### 2.12. Western Blot

The total proteins from the cells were extracted by RIPA buffer supplemented with phosphatase and protease inhibitors. Subsequently, equal amounts of cell extracts were electrophoreted in 10% gradient SDS–PAGE gels, and transferred onto PVDF membranes (Millipore, Chicago, MA, USA). The membranes were then blocked with 5% BSA in TBST for 1 h at room temperature and incubated with primary antibodies against Bcl-2, Bcl-xl, Mcl-1, GLUT1, HK2, PKM2, LDHA, GAPDH and α-tubulin (Cell Signaling Technology, Essex County, MA, USA) overnight at 4 °C. Following this, the membranes were incubated with peroxidase-conjugated goat anti-rabbit or anti-mouse secondary antibodies for 1 h at room temperature. The immunoreactive bands were measured with an enhanced chemiluminescence (ECL) kit (Vazyme, Nanjing, China), and monitored using a chemiluminescent imaging system (Tanon Science & Technology, Shanghai, China).

### 2.13. Xenotransplantation Experiments

For the experimental metastasis studies, B-NDG mice were administered tail vein injections of luciferase-labeled Jurkat cells, either β-catenin-sg or control. Subsequently, the mice were injected 150 mg/kg D-luciferin intraperitoneally and imaged by the Lumina in vivo imaging system (IVIS). After three weeks, the mice were euthanized, and their livers and spleens were excised, imaged, and quantified utilizing the IVIS Spectrum in vivo imaging system. All animal experiments received approval from the Animal Research Ethics Committee at the Guangzhou Institute of Biomedicine and Health (Approval Code:A5748-01). Furthermore, all procedures were conducted in strict accordance with the pertinent guidelines and regulations.

### 2.14. Transcriptome Sequencing

Total RNA was extracted from both β-catenin knockout and control cells, and then purified utilizing the RNeasy Mini Kit following the manufacturer’s protocol. Subsequent analyses, including Gene Ontology (GO) and Gene Set Enrichment Analysis (GSEA), were conducted employing the cluster Profiler v4.2.0 package.

### 2.15. Statistical Analyses

All quantitative data were analyzed using GraphPad Prism Ver. 8.0 (GraphPad Software Inc., San Diego, CA, USA) and are presented as the means ± standard deviations (SDs) of three independent experiments. Statistical significance was assessed using unpaired t-test analysis for comparisons between the two groups. A *p*-value of less than 0.05 was considered to indicate statistical significance.

## 3. Results

### 3.1. β-Catenin Deficiency Restrained Cell Proliferation and Promoted Apoptosis

**Figure 1:**
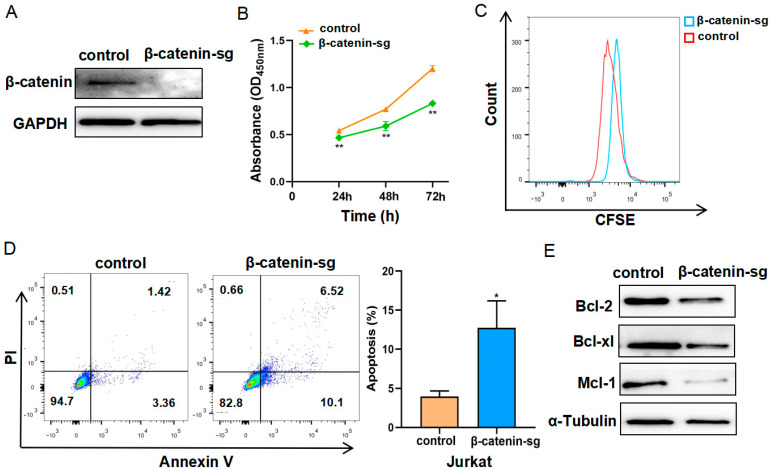
β-catenin deficiency restrained cell proliferation and promoted apoptosis. (**A**) Western blot analysis of β-catenin knockout efficiency in Jurkat cells. (**B**) The cell proliferation rates of control cells and β-catenin knockout cells were determined using the CCK-8 reagent. (**C**) The effect of β-catenin on cell proliferation was detected using the CFSE staining assay. (**D**) Apoptosis analysis was determined via flow cytometry using Annexin V/PI staining, and the proportion of total apoptotic cells was assessed. (**E**) Western blot analysis of Bcl-2, Bcl-xl and Mcl-1 in control and β-catenin knockout cells. α-tubulin served as a control. * *p <* 0.05, ** *p <* 0.01.

To investigate the role of β-catenin in T-ALL, we generated β-catenin knockout cells utilizing the CRISPR-Cas9 technique. The efficiency of β-catenin deletion was confirmed through Western blot analysis, which demonstrated a significant reduction in β-catenin protein levels (Figure 1A). Subsequently, the cells were cultured in RPMI 1640, 10% FBS for 0, 24, 48, and 72 h. Cell proliferation was detected using the CCK8 assay and CFSE staining assay. The results indicated a decrease in cell growth rates in the β-catenin knockout cells (Figure 1B,C). We analyzed cell apoptosis utilizing flow cytometry with PI and annexin V staining. The results demonstrated an elevated proportion of apoptotic cells in a β-catenin knockout group relative to the control group (Figure 1D). Furthermore, Western blot analysis was employed to assess apoptosis-related proteins. The knockout of β-catenin resulted in a significant decrease in the expression of Bcl-2, Bcl-xl and Mcl-1 (Figure 1E). Collectively, these findings show that β-catenin deletion suppresses cell proliferation and promotes apoptosis in T-ALL cells.

### 3.2. RNA-Seq Analysis Revealed That β-Catenin Might Regulate T-ALL Metabolism

**Figure 2:**
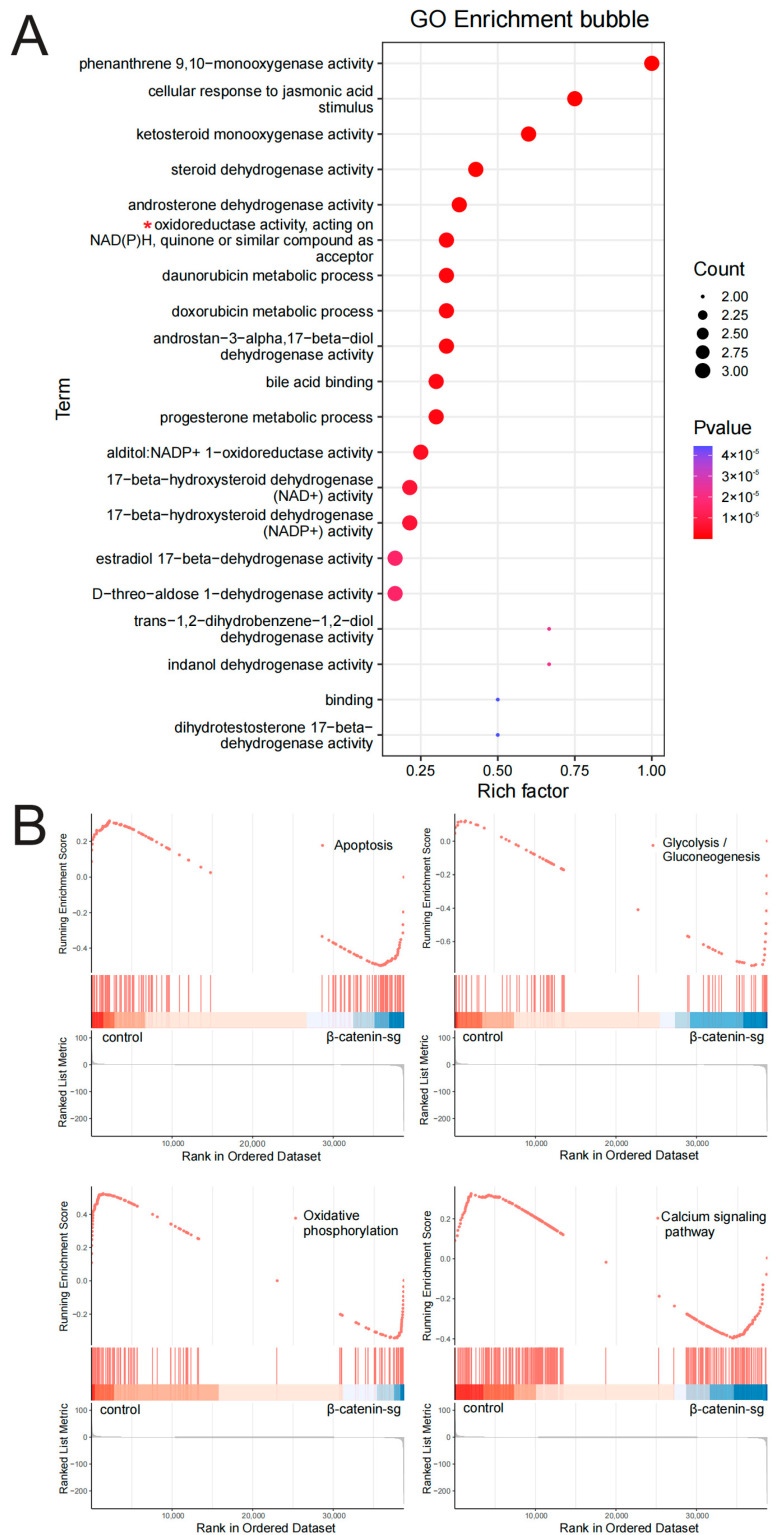
RNA-seq analysis revealed that β-catenin might regulate T-ALL metabolism. (**A**) Differential expressed genes from β-catenin sg and control cells were subjected to Gene Ontology (GO) analysis for biological processes (BPs), cellular components (CCs), and molecular functions (MFs). (**B**) Gene Set Enrichment Analysis (GSEA) was performed.

To elucidate the role of β-catenin in cell growth, proliferation, and metabolism of T-ALL cells, we investigated the global transcriptional profiles between β-catenin knockout and the control group. Gene Ontology (GO) functional enrichment analysis demonstrated that β-catenin is implicated in redox reactions (Figure 2A). Gene Set Enrichment Analysis (GSEA) indicated that β-catenin participates in the modulation of cell apoptosis, glycolysis, oxidative phosphorylation and calcium signaling pathway (Figure 2B). These results confirm that β-catenin plays a regulatory role in cell apoptosis and glycometabolism.

### 3.3. β-Catenin Deficiency Inhibited the Glycolysis of T-ALL Cells

**Figure 3:**
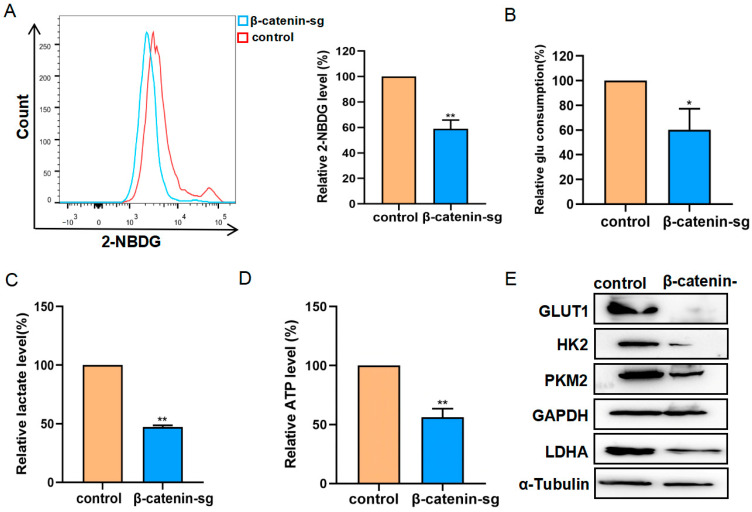
β-catenin deficiency inhibited the glycolysis of T-ALL cells. (**A**) Glucose uptake was detected by flow cytometry using 2-NBDG staining. (**B**,**C**) Glucose consumption and lactate production in the control and β-catenin sg cells were determined. (**D**) Relative ATP levels in the control and β-catenin sg cells were determined normalized with protein concentration. (**E**) Western blot analysis of GLUT1, HK2, PKM2, GAPDH and LDHA. α-tubulin served as a control. * *p <* 0.05, ** *p <* 0.01.

To further elucidate the role of β-catenin in regulating glycometabolism, we employed a 2-NBDG probe to detect glucose uptake, which exhibited a significant reduction following β-catenin knockout (Figure 3A). Additionally, glucose consumption, lactate release, and ATP generation were all diminished in β-catenin knockout cells compared to control cells (Figure 3B–D). Glycolysis-associated proteins were analyzed by Western blot. The results demonstrated the downregulation of glycolysis-related proteins, including GLUT1, HK2, LDHA, and PKM2 in β-catenin knockout cells relative to control cells (Figure 3E). These findings confirm that the knockout of β-catenin may influence glycolysis through the modulation of glycolytic protein expression.

### 3.4. β-Catenin Deficiency Induced Mitochondrial Impairment in T-ALL Cells

**Figure 4:**
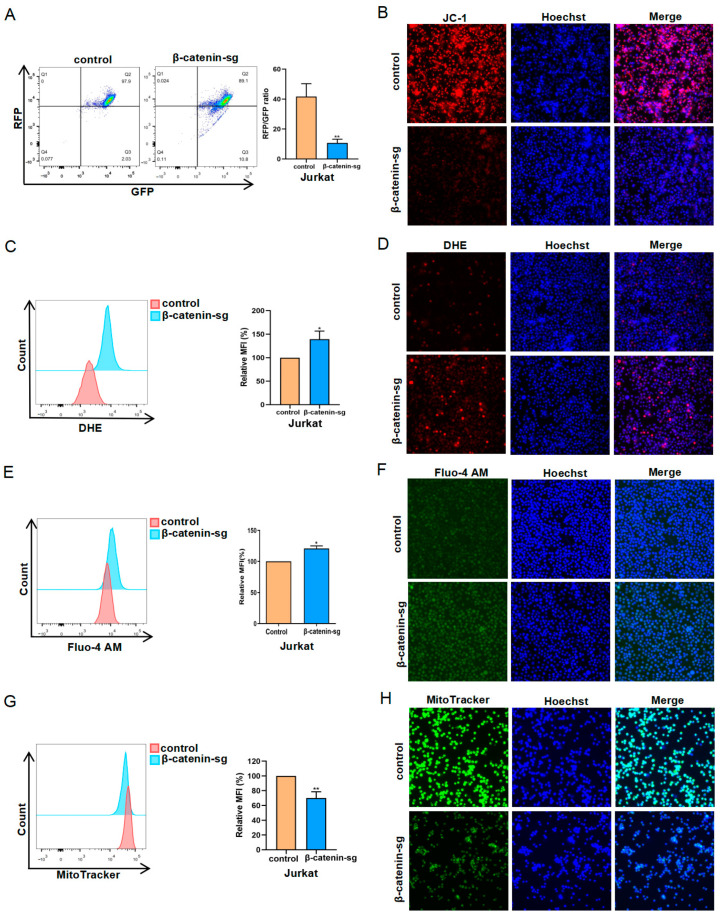
β-catenin deficiency induced mitochondrial impairment in T-ALL cells. (**A**) The mitochondrial membrane potential was determined using JC-1 staining by flow cytometry and (**B**) cells were photographed by fluorescence microscope. (**C**) The ROS level was quantified using DHE staining by flow cytometry and (**D**) cells were photographed by fluorescence microscope. (**E**) The cellular calcium levels were detected using Fluo-4 AM probe by flow cytometry and (**F**) cells were photographed by fluorescence microscope. (**G**) The mitochondrial mass was determined using MitoTracker staining by flow cytometry and (**H**) cells were photographed by fluorescence microscope (40×). * *p <* 0.05, ** *p <* 0.01.

To investigate mitochondrial functional alteration following β-catenin knockout, we employed the JC-1 probe to assess mitochondrial membrane potential and MitoTracker to evaluate mitochondrial mass. As illustrated in (Figure 4A,B), β-catenin knockout led to a decrease in the mitochondrial membrane potential. The disruption of mitochondrial membrane potential contributes to elevated superoxide levels, given that mitochondria are the primary organelles involved in oxidative phosphorylation (OXPHOS) [12]. The levels of ROS were found to be elevated following the deletion of β-catenin (Figure 4C,D). Mitochondria serve as the primary organelles responsible for regulating calcium dynamics [13]. The Fluo-4 AM probe was employed to assess intracellular calcium levels. Following the knockout of β-catenin, intracellular calcium concentrations were significantly increased, resulting in a disruption of calcium homeostasis within the cells (Figure 4E,F). Correspondingly, mitochondrial mass was also diminished in β-catenin knockout cells (Figure 4G,H). Consequently, β-catenin deficiency induced a reduction in mitochondrial mass and mitochondrial membrane potential, ROS generation and disruption of calcium equilibrium in leukemic cells.

### 3.5. β-Catenin Promoted Organ Infiltration of T-ALL Cells

**Figure 5:**
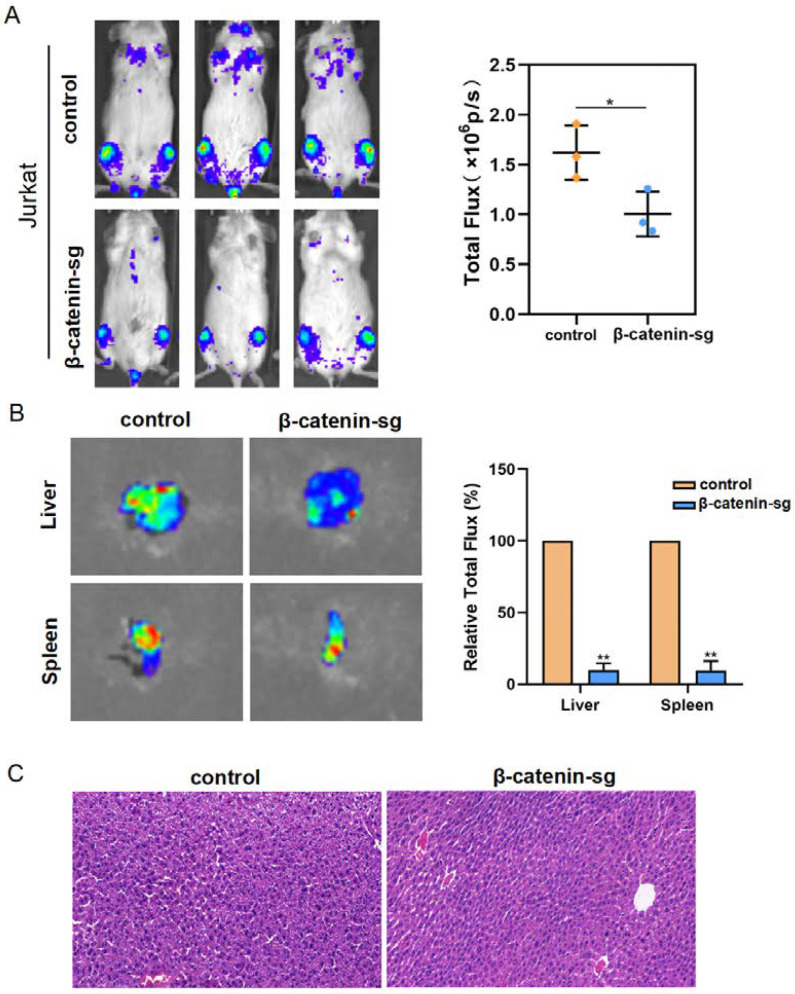
β-catenin promoted organ infiltration of T-ALL cells. (**A**) Luciferase-labeled B-NDG mice were injected intraperitoneally with D-luciferin and imaged after 3 weeks. (**B**) Representative bioluminescent images of the isolated livers. (**C**) Hematoxylin and eosin (HE) staining of the liver in the xenografts were performed (100×). * *p* < 0.05, ** *p* < 0.01.

To verify whether β-catenin stimulates leukemia cell infiltration, Jurkat cells expressing vector-luc or β-catenin sg-luc were injected via tail vein into immunodeficient mice. The whole-animal imaging analysis displays that the bioluminescence signal intensity decreased in the β-catenin deletion group compared to the vector group (Figure 5A). Moreover, the liver metastatic abilities were greatly decreased in the β-catenin sg group (Figure 5B,C). In all, these findings reveal that β-catenin promoted T-ALL cell infiltration in vivo.

## 4. Discussion

Numerous studies have established β-catenin as a pivotal oncogenic driver implicated in various phases of leukemogenesis and leukemic stem cells [14,15]. Our prior research revealed a direct correlation between β-catenin activity and leukemia-initiating cells in T-ALL [16]. In gastric and breast cancer cells, suppression of the Wnt/β-catenin signaling pathway remarkably inhibited cell viability and promoted apoptosis by increasing apoptotic proteins [17,18]. Here, we also demonstrated that β-catenin silencing could inhibit cell proliferation and promote apoptosis in T-ALL cells. Nevertheless, the role of β-catenin in the metabolism of T-ALL remains poorly understood.

Cellular metabolism is closely related to tumorigenesis and development. Glycolysis is a major energy metabolic pathway for tumor cells, and has been claimed as extensively important to cell growth and apoptosis [19,20]. Leukemia development depends upon high levels of glycolysis, which may provide a therapeutic target for AML [21,22]. Moreover, elevated glycolysis levels are also found in T-ALL [23,24,25]. However, little is known about the effects of β-catenin in T-ALL glycolysis. Bioinformatics analysis of an RNA-seq dataset revealed that β-catenin participates in the modulation of the redox reaction, cell apoptosis, glycolysis, oxidative phosphorylation and calcium signaling pathway. These data reveal that β-catenin may play an important role in T-ALL cell metabolic processes.

We determined that β-catenin knockout decreased glycolysis, including reduced glucose uptake, lactate, glucose consumption and ATP levels in T-ALL cell lines. These data indicate a previously unexpected role of β-catenin in T-ALL. Perinatal cardiomyocyte research indicated an inhibition of glycolysis following β-catenin deletion in in vitro and in vivo models [26]. Lactate dehydrogenase inhibitors reduced cell glycolysis and inhibited tumor growth [27], which implied that the reduced ATP and lactate levels might cause the inhibited growth of T-ALL cells. The key glycolysis enzymes play crucial roles in the cell proliferation, infiltration, and drug resistance of tumor cells. We observed downregulation of the expressions of HK2, GLUT1, PKM2, and LDHA in β-catenin knockout cells. This suggests that β-catenin deletion inhibited cellular glycolysis by reducing glycolysis-related protein expression, but the regulatory mechanism needs to be explored.

Mitochondria are the main organelles that provide energy for the production of ATP [28] and are involved in essential cellular processes, for instance, cell apoptosis, metabolite synthesis, Ca^2+^ homeostasis, and ROS production [29]. In addition, mitochondria are vital for the maintenance of hematopoietic stem cells (HSCs) and their normal function [30]. Mitochondrial dysfunction not only leads to impaired energy production, but also results in pathological conditions, including cancer [31,32]. However, the accurate role of β-catenin in regulating mitochondrial functions remains conflictive. Previous studies show that β-catenin has either stimulatory or inhibitory influences on mitochondria function [33,34]. Meanwhile, in perinatal cardiomyocytes, both the deletion and inhibition of β-catenin reduced the mitochondrial number, caused mitochondrial disintegration, and induced mitochondrial function impairment [26].

In this study, we observed decreased mitochondrial membrane potential and enhanced ROS levels in β-catenin-deleted T-ALL cells. Excessive ROS levels might induce the disruption of the mitochondrial membrane structure, resulting in decreased mitochondrial membrane potential, which induces mitochondrial impairment. Mitochondria serve as important organelles for the regulation and storage of calcium [35]. In Baran N’s study, they characterized the metabolic features of T-ALL and elucidated that targeting metabolic reprogramming in T-ALL is an effective way to control disease burden in vivo [36]. Metabolic reprogramming might become a promising avenue for the investigation of new therapeutic interventions in relapsed T-ALL.

In acute myelogenous leukemia (AML) mouse models, β-catenin participates in the self-renewing of leukemia stem cells (LSCs) [37]. In human leukemias and mouse models of T-ALL, β-catenin is expressed in high levels in LSCs, highlighting that β-catenin is involved in T-ALL maintenance and progression [6,15,38]. In breast cancer CSCs, β-catenin mRNA is significantly upregulated and correlates with the self-renewal function of CSCs [39]. In addition, in endometrial carcinoma, β-catenin may be a significant tumorigenic factor and provide an approach to assess early-stage tumor recurrence [40]. In consistency with these findings, our data also showed that β-catenin promoted T-ALL progression in vivo and may provide a more effective treatment for T-ALL. The detailed mechanism needs further investigation using various mouse models. Our study indicates that targeting β-catenin may hold significant therapeutic potential for relapsed T-ALL patients.

## 5. Conclusions

**Figure 6:**
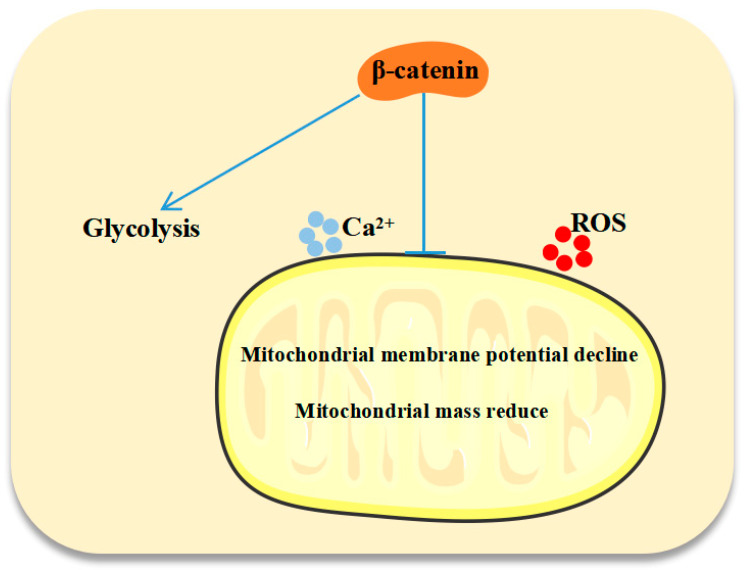
The schematic diagram displays the mechanism of β-catenin-regulating glycolytic and mitochondrial function in T-ALL.

In conclusion, our research confirms the critical role of β-catenin in the modulation of T-ALL metabolism, as indicated by the decreased glycolysis and impaired mitochondrial function after β-catenin silencing (Figure 6). Further proteomic analysis of β-catenin deficiency could elucidate its involvement in metabolism. The metabolic reprogramming process is a potential therapeutic target, and our study provides a promising strategy for T-ALL treatment. Future T-ALL treatment strategies will perhaps include β-catenin as well as metabolic-reprogramming-targeting drugs.

## Data Availability

Data will be made available on request.
