# Correction: Zhang et al. β-Catenin Regulates Glycolytic and Mitochondrial Function in T-Cell Acute Lymphoblastic Leukemia. *Biomedicines*
**2025**, *13*, 292

**DOI:** 10.3390/biomedicines13092064

**Published:** 2025-08-25

**Authors:** Ling Zhang, Yu Zhao, Shuoting Wang, Jian Zhang, Xiaohui Li, Shuangyin Wang, Taosheng Huang, Jinxing Wang, Jiajun Liu

**Affiliations:** 1Department of Hematology, The Third Affiliated Hospital of Sun Yat-sen University, Guangzhou 510630, China; zhangl389@mail.sysu.edu.cn (L.Z.); wangsht39@mail2.sysu.edu.cn (S.W.); lixxiaoh0831@163.com (X.L.); 2Department of Hematology, The Third Affiliated Hospital of Southern Medical University, Guangzhou 510630, China; zhyhg0203@163.com (Y.Z.); wangshuangyin1998@163.com (S.W.); 3Guangzhou Institutes of Biomedicine and Health, Chinese Academy of Sciences, Guangzhou 510530, China; shuifen1983@163.com; 4The Medicine and Biological Engineering Technology Research Center of the Ministry of Health, Guangzhou 510663, China; tsh_huang@sina.cn; 5Department of Pathology Technique, Guangdong Medical University, Dongguan 523808, China

## Text Correction

The apoptosis experiment in the updated reference did not involve the detection of caspase-9. The methods in the original publication [1] did not involve the detection of caspase-3/9. A correction has been made to delete “, for instance, caspase-3/9” in the Discussion Section on paragraph 1.

## References

In the original publication [1], citation reference 17 has been retracted; the authors unintentionally cited this retracted reference. The authors updated the retracted citation reference. The new citation reference appears below. The authors state that the scientific conclusions are unaffected. This correction was approved by the Academic Editor. The original publication has also been updated.

17.Bhattacherjee, D.; Raina, K.; Mandal, T.K.; Thummer, R.P.; Bhabak, K.P. Targeting Wnt/β-catenin signaling pathway in triple-negative breast cancer by benzylic organotrisulfides: Contribution of the released hydrogen sulfide towards potent anti-cancer activity. *Free Radic. Biol. Med.*
**2022**, *191*, 82–96. https://doi.org/10.1016/j.freeradbiomed.2022.08.029.
